# Increased Cellular Uptake of ApoE3- or c(RGD)-Modified Liposomes for Glioblastoma Therapy Depending on the Target Cells

**DOI:** 10.3390/pharmaceutics16091112

**Published:** 2024-08-23

**Authors:** Larissa J. Lubitz, Moritz P. Haffner, Harden Rieger, Gero Leneweit

**Affiliations:** 1 ABNOBA GmbH, 75223 Niefern-Öschelbronn, Germany; 2 Carl Gustav Carus-Institute, 75223 Niefern-Öschelbronn, Germany; 3 Department of Chemical and Process Engineering, Institute of Mechanical Process Engineering and Mechanics, Karlsruhe Institute of Technology, 76131 Karlsruhe, Germany

**Keywords:** nervous system disease, lipids, peptide, protein, pharmaceutical preparations, cells, therapeutics, cancer

## Abstract

As effective treatment of glioblastoma is still an unmet need, targeted delivery systems for efficient treatment are of utmost interest. Therefore, in this paper, surface modifications with a small peptide c(RGD) or physiological protein (ApoE3) were investigated. Cellular uptake in murine endothelial cells (bEnd.3) and different glioma cells (human U-87 MG, rat F98) was tested to elucidate possible differences and to correlate the uptake to the receptor expression. Different liposomal formulations were measured at 1 and 3 h for three lipid incubation concentrations. We calculated the liposomal uptake saturation *S* and the saturation half-time *t*_1/2_. An up to 9.6-fold increased uptake for ApoE3-modified liposomes, primarily in tumor cells, was found. Contrarily, c(RGD) liposomes showed a stronger increase in uptake in endothelial cells (up to 40.5-fold). The uptake of modified liposomes revealed enormous differences in *S* and *t*_1/2_ when comparing different tumor cell lines. However, for ApoE3-modified liposomes, we proved comparable saturation values (~25,000) for F98 cells and U-87 MG cells despite a 6-fold lower expression of LRP1 in F98 cells and a 5-fold slower uptake rate. Our findings suggest that cellular uptake of surface-modified liposomes depends more on the target structure than the ligand type, with significant differences between cell types of different origins.

## 1. Introduction

Approximately 80% of primary malignant tumors of the central nervous system are glioblastomas, making these the most common malignant brain tumor in adults [1]. With an incidence of 3.26 per 100,000 individuals annually in the US, this number is rising due to the aging population, pollution, and advancements in diagnosis [1,2]. Despite advanced diagnostics and treatments, the prognosis remains poor, with a 5-year survival rate of around 5% and median survival of 8 to 15 months [1,3].

Various strategies have been devised to overcome the blood–brain barrier (BBB) in treatment, including chemical or physical modulation to increase permeability [4] and alternative administration routes like intranasal or local administration [5,6,7]. However, the development of efficient drug delivery systems modified with ligands for targeted transport to the BBB or glioma cells is of paramount importance, as this allows the integrity of the BBB to be maintained [8].

The BBB, a major barrier for drug transport into the brain [9], is primarily a biochemical rather than physical barrier [10], protecting the brain from pathogens and maintaining its homeostasis [11,12]. It blocks about 98% of small molecules and 100% of large macromolecules [12]. Lipid solubility, charge, hydrogen bonding, ionization profile, and molecular weight are important for substance transport across the BBB [13]. Specific endothelial cells connected by tight junctions [11] limit therapeutic permeability and transport [14,15]. Different pathways across the BBB include the paracellular aqueous route, the transcellular lipophilic route, adsorptive transcytosis, receptor-mediated transport (RMT), and carrier-mediated transport [16].

The RMT and its receptors at the BBB represent attractive structures for developing targeted drug delivery systems, acting as a Trojan horse for delivering therapeutics into the brain. RMT’s active internalization without size limitation makes functionalized nanocarriers’ interaction with BBB targets particularly interesting.

A large number of receptors are prominent at the BBB, such as insulin-like growth factor receptors (IR), type 1 transferrin receptors (TfR1), leptin receptors (LEPR), and low-density lipoprotein receptors and LDL-related receptor protein 1 (LRP1). Andersen and Willnow (2006) were able to show that LRP1 is expressed at a significantly higher rate in neurons and is also localized in the abluminal endothelial cell membrane [17,18], in contrast to the LDL receptor, which is also found in the liver. Since cancer cells have a high demand for cholesterol as a result of increased cell membrane synthesis, LRP1 represents an attractive target for the targeted transport of drugs across the BBB.

Apolipoprotein E is crucial for the regulation of cholesterol homeostasis in the peripheral circulation, but its role in the brain appears to involve not only cholesterol transport but also the intercellular exchange of metabolites between neurons and glial cells, which is required for the maintenance of healthy brain tissue [19]. Within the ApoE family, the ApoE3 isoform appears to play an important role in nanoparticle-mediated drug transport across the BBB [20], as it is the most abundant isoform, comprising around 50–70% of all ApoE [21]. ApoE3 is used as a strategy to overcome the BBB for many different indications. In addition to brain tumors, neurodegenerative diseases such as Alzheimer’s [22] and brain-tumor-related epilepsy [23] have been studied.

An increase in BBB permeability by modification of polymeric or albumin nanoparticles [20,24,25] or solid lipid nanoparticles [26,27,28] with ApoE3 has already been demonstrated. No differences in uptake behavior between covalently and adsorptively bound apolipoprotein E or A1 were recorded by Kreuter et al. (2007) and Petri et al. (2007) [29,30].

In addition to the BBB as a physiological barrier for the effective transport of drugs into the brain, another obstacle is the infiltrative nature of the glioblastoma itself. Thus, strong vascularization of a highly malignant tumor is important for rapid growth [31,32]. Vascularization involves early cooptation of normal cerebral blood vessels and angiogenesis of the tumor region [33,34]. The treatment of glioblastoma is currently attempted by a surgical therapy, supplemented by radiotherapy and chemotherapy [35,36]. However, this combined approach is not able to eliminate all glioblastoma cells and cannot destroy the abnormal vessels around the glioblastoma tissue, which leads to frequent tumor recurrence [37]. Thus, the α_V_β_3_ receptor from the integrin receptor family is one of the most promising targets for the targeted transport of chemotherapeutics, as it is overexpressed in the actively proliferating endothelium of tumor tissue [38,39]. A large number of in vitro studies in recent decades have already shown that modification of nanocarriers with the cyclic tripeptide c(RGD), which can bind the integrin receptor, is a promising option for treatment of a variety of tumors [40]. However, surface modification of DDS with c(RGD) is not only of interest for the targeted therapy of tumors. Cardiovascular and neurodegenerative diseases as well as strokes represent further potential therapeutic areas [41].

Inorganic nanoparticles [42,43,44], as well as phospholipid nanoparticles such as liposomes [45,46], nanogels [47,48], and polymers [49,50,51], have already shown in vitro or in vivo that they can overcome the BBB via surface modifications. However, none of them have been tested with active targeting ligands, which is needed to improve therapeutic outcomes. Since no nanoparticulate transport system with a coating for active targeting has yet been commercially approved, there is a clear need for further pre-clinical trials to develop the most promising targeting. Due to their mature production method, high membrane permeability, and simple surface modification methods, liposomes are of paramount importance [52,53,54]. In addition, liposomes offer further advantages, such as non-toxicity, biocompatibility, and biodegradability [55]. They represent an effective, non-invasive method of transporting hydrophilic, lipophilic, or amphiphilic drugs across the BBB through surface modification. These surface modifications can be accomplished using a variety of approaches. For both ApoE3 and c(RGD), the ligand can be adsorbed [56,57] or covalently bound via a maleimide-thiol bond [20,58]. A covalent bond using the click chemistry of maleimide and a thiol-group offers many advantages, such as rapid kinetics, the absence of additional additives for the reaction, and the prevention of radical formation. In addition, the covalent bond of the ligand allows a precise quantification of the surface-bound ligand after a purification step [59].

The present work deals with the modification of liposomes with a peptide or a protein as a ligand. The dual focus was on both apolipoprotein E3 as a macromolecular endogenous structure and the cyclic tripeptide of arginine, glycine, and aspartic acid (c(RGD)). Both ligands were covalently bound to liposomes and their influence on cellular uptake in cerebral endothelial cells and glioma cells was investigated to elucidate any time or concentration-related dependencies and to further correlate with the receptor expression on the cell surface. In addition, this work aims to answer the question of whether there are differences in cellular uptake behavior for the same cell types (glioma) that are of different origins.

## 2. Materials and Methods

### 2.1. Materials

LIPOID GmbH (Ludwigshafen, Germany) supplied the phospholipids DPPC and DSPE-mPEG, while Biopharma PEG Scientific Inc. (Watertown, NY, USA) provided DSPE-PEG5k-Mal and DPSE-PEG5k-c(RGDyk). Sodium chloride, cholesterol, ethanol, and L-histidine were obtained from Carl Roth GmbH (Karlsruhe, Germany).

### 2.2. Preparation of Liposomes

Thin-film hydration was used to prepare the liposomes, followed by membrane extrusion. To achieve this, stock solutions of the following components were prepared in ethanol: DPPC, cholesterol, DSPE-MPEG, DSPE-PEG5k-Mal, and DSPE-PEG5k-c(RGDyk). These solutions were then rotated into thin lipid films in the intended combinations using a rotary evaporator.

Using a 10 mM histidine buffer containing 0.3 osmol/L sodium chloride at a pH of 7.4, the lipid film was rehydrated to a final total lipid concentration of 20 mM. The extrusion of liposomes through track-etched polycarbonate membranes (Whatman™, Cytiva, Marlborough, MA, USA) was carried out in two steps using the Lipex^®^ extruder (Northern Lipids Inc., Burnaby, BC, Canada): (1) five times through a 400 nm pore-size membrane, followed by (2) twenty times through a membrane with a 100 nm pore. Argon was used to pressurize the extrusion process, with pressures ranging from 10 bar to 25 bar.

**Figure 1:**
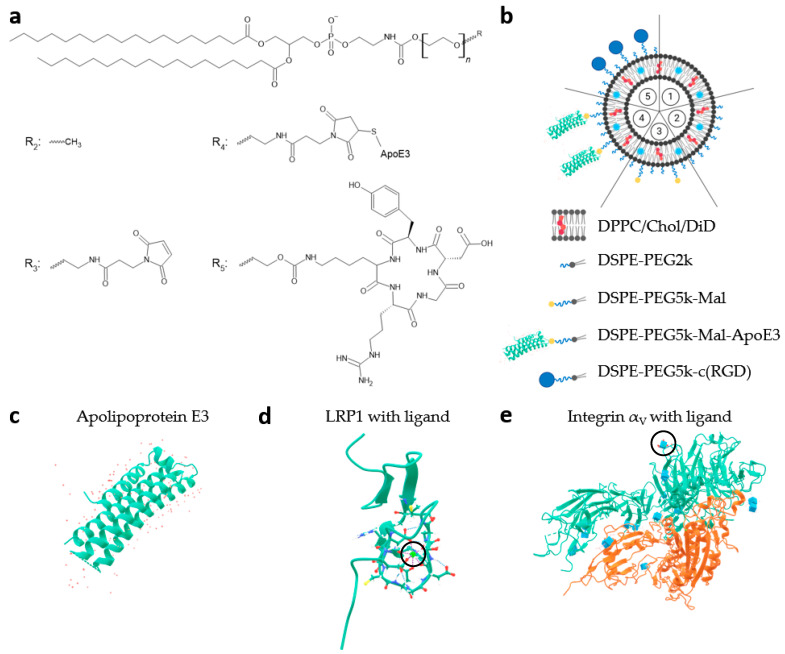
Schematic overview of structures, different liposomal formulations, and tertiary protein structures. (**a**) Displays different distal ends (R_2_ to R_5_) of the DSPE-PEG5k in liposomal formulations 2 to 5 (as shown in scheme (**b**)); (**b**) illustrates a schematic overview of the 5 liposomal formulations; (**c**) shows the tertiary structure of apolipoprotein E3 (ApoE3; Protein Data Base (PDB) code: 1NFN); (**d**) low-density lipoprotein receptor-related protein 1 (LRP1) with bound ligand (PDB code: 1CR8); and (**e**) shows crystal structure of the extracellular segment of integrin α_V_β_3_ with bound ligand (PDB code: 1JV2). The black circles mark the ligand binding site.

Figure 1a depicts the structure of the different distal ends of the DSPE-PEG5k, whereas Figure 1b shows a schematic representation of the liposomal composition, which is detailed in Table 1. Figure 1c illustrates the protein structures of apolipoprotein E3, which was covalently bound to liposomes as a ligand for the LRP1 receptor (Figure 1d). Figure 1e displays the integrin α_V_β_3_ receptor as a target for liposomal bound c(RGD).

### 2.3. Surface-Modification of Liposomes with Apolipoprotein E3

#### 2.3.1. Thiolation of Apolipoprotein E3

To facilitate the conjugation of apolipoprotein E3 (enQuire™ Bio LLC, Littleton, CO, USA) following liposome production, DSPE-PEG5k-Mal was added to liposomes at a concentration of 0.1 mol%. In the following, this process will be referred to as “post-conjugation”. The conjugation of apolipoprotein E3 (ApoE3) required a prior modification by Traut’s reagent (2′Iminothiolan, Santa Cruz Biotechnology, Dellas, TX, USA), which resulted in the thiolation of existing primary amine groups on, for example, lysine side chains. Thiolation was achieved by adding a 50-fold molar excess of Traut’s reagent. The reagent was used at a concentration of 50 mg/mL in PBS with 5 mM EDTA (Carl Roth GmbH & Co. KG, Karlsruhe, Germany). After incubation for 2 h on the rotary wheel under an argon atmosphere, purification was performed using Zeba™ Spin Column 7 kDa MWCO (Thermo Fisher Scientific, Waltham, MA, USA). An Ellman’s detection with 5,5′-Dithiobis-(2-nitrobenzoic acid) (DNTB, Santa Cruz Biotechnology, Dallas, TX, USA) for the detection of thiol groups was performed according to the manufacturer’s instructions. A repetition of this assay was performed after sterile filtration of the modified liposomes to confirm the conjugation of thiolated ApoE3 to maleimide-functionalized liposomes, termed “Mal” in the following.

#### 2.3.2. Post-Conjugation of Apolipoprotein E3 to Liposomes

In order to facilitate the conjugation of apolipoprotein E3 (enQuire™ Bio LLC, Littleton, CO, USA) following liposome production, DSPE-PEG5k-Mal was added to liposomes at a concentration of 0.1 mol%. In the following, this process will be referred to as “post-conjugation”. To post-conjugate ApoE3 (2 mg/mL in PBS), 0.1 mol% of DSPE-PEG5k-Mal was added to the liposomes. The maleimide-containing liposomes were conjugated by incubating a 2 mg/mL solution of ApoE3 with the addition of a 20-fold molar excess of Tris-(2-carboxyethyl)-phosphine hydrochloride (TCEP; 5 mg/mL in PBS) at a mass ratio of 1.5:1 (equivalent to a molar ratio of 8.7:1) on the spinning wheel for 24 h at room temperature. Following a 24 h conjugation period, unreacted ApoE3 was removed by dialyzing the crude liposomal product in a Spectra/Por^®^ Biotech CE (Repligen, Waltham, MA, USA) tube with a molecular weight cut-off of 100 kDa. The dialysis medium was added outside the dialysis membrane in a 300-fold excess of the sample and changed after 2, 4, and 24 h. It was made of the same buffer utilized for the preparation of liposomes. Finally, a syringe filter with a pore size of 0.22 µm was used to filter each liposomal formulation in a sterile manner.

### 2.4. Characterization of Liposomes

Dynamic light scattering was used to measure the liposomes’ mean hydrodynamic diameter (Z-Ave) and polydispersity index (PdI) after extrusion and final sterile filtration. The liposomal formulations were measured using the ZetaSizer ZS90 (Malvern Instruments, Worcestershire, UK) to characterize them according to their size and polydispersity index. Three measurements of each sample, each consisting of five single runs, were made. 

For a duration of four weeks, DLS measurements were performed weekly for all liposomal samples, which were kept at 4 °C to ascertain physical liposomal stability.

The Human ApoE ELISA ^BASIC^ Kit (Mabtech AB, Nacka Strand, Sweden) was used to determine the amount of ApoE3 on the liposomal surface.

Using the LabAssay^TM^ Cholesterol Assay Kit (FujiFilm Wako Chemicals Europe GmbH; Neuss, Germany), the cholesterol content of the liposomes was determined to calculate the total lipid concentration.

### 2.5. In Vitro Studies

#### 2.5.1. Cell Culture and Reagents

Murine bEnd.3 brain endothelial cells were obtained from the European Collection of Animal Cell Cultures (96091929; Sigma-Aldrich, Saint-Louis, MI, USA). The human U-87 MG GMB cells (Uppsala 87 Malignant Glioma; HTB-14) as well as the rat F98 cells (ATCC CRL-2397) were obtained from the American Type Culture Collection (Manassas, VA, USA). Dulbecco’s modified Eagle media with a 4.5 g/L glucose (Carl Roth GmbH & Co. KG, Karlsruhe, Germany) content was used to cultivate all three cell lines at 37 °C and 10% CO_2_.

In order to supplement the culturing medium, 10% (*v*/*v*) FBS, 100 U/mL penicillin, 0.1 mg/mL streptomycin, and 0.1% (*v*/*v*) non-essential amino acids (NEAA) were applied. All mentioned supplements were purchased from PAN-Biotech GmbH (Aidenbach, Germany).

#### 2.5.2. Cell Staining Assay

After being seeded with 6 × 10^4^ cells/well in 24-well plates, the cells were incubated for 24 h at 37 °C and 10% CO_2_. The culture media utilized was DMEM with high glucose concentration of 4.5 g/L. The cells were detached using Accutase^®^ (VWR International, Avantor Group, Karlsruhe, Germany) following a wash step with Dulbecco’s phosphate saline (DBPS, Carl Roth GmbH & Co. KG, Karlsruhe, Germany). 

Following the cells’ transfer to a FACS tube, a cell pellet was formed by centrifuging for five minutes at 150× *g*, which then was resuspended in FACS buffer (DPBS plus 5% (*v*/*v*) FBS). Afterward, a blocking step to inhibit non-specific Fc-mediated antibody interactions by incubating the cells with anti-mouse (Ab93) or anti-rat CD16/CD32 (D34-485) antibodies or human Fc-block binding inhibitor (Fc1) (BD Biosciences Inc., Franklin Lakes, NJ, USA) for 20 min at room temperature was performed.

Upon the removal of the supernatant, the cells were incubated on a shaker at room temperature for 20 min with 4% (*v*/*v*) paraformaldehyde (Carl Roth GmbH & Co. KG, Karlsruhe, Germany). After two steps of washing in FACS buffer, the cells were permeabilized by adding two to three drops of 100% ice-cold methanol dropwise and then being incubated for five minutes at room temperature on a shaker.

After three additional steps of washing with FACS buffer, the cells were stained using 100 µL of a 1:200 dilution of either the phycoerythrin (PE)-coupled LRP1 antibody (A2MRα-2) or the PE-coupled integrin α_V_ antibody (A-11). Both staining antibodies were obtained from Santa Cruz Biotechnology (Dellas, TX, USA) in FACS buffer. For thirty minutes, the incubation process was conducted in a refrigerator with light protection. Subsequently, the cells were resuspended in 500 µL of FACS buffer after undergoing three rounds of washing. A total of 10^4^ single-cell events were measured with the flow cytometer (BD LSR II, BD Biosciences Inc., Fraklin Lakes, NJ, USA) to obtain the mean fluorescence intensity (MFI).

#### 2.5.3. Liposomal Uptake Assay

At a density of 3 × 10^4^ cells/well in 48-well plates, the cells were cultured for 24 h at 37 °C and 10% CO_2_ in DMEM with 4.5 g/L glucose. Afterward, 90% of the well volume was refilled with culture medium as the media was aspirated. The liposomal formulations to be evaluated were then added, making up 10% of the well capacity, to reach the final lipid concentrations of 100, 500, and 1000 µM. The required dilutions of the liposomal formulations to a lipid concentration of either 10 mM, 5 mM or 1 mM were made ahead using the culture medium for dilution. The experiment was conducted in an incubator with 10% CO_2_ and 37 °C for 1 or 3 h.

The cellular uptake of the liposomes was stopped by aspiration of the media and application of ice-cold DPBS to the cells. In order to detach the cells, trypsin-EDTA (BioWest S.A.S, Nuaillé, France) was diluted 1:10 with DPBS and added to the wells. The trypsin-EDTA was inactivated with cold FACS buffer. Following their transfer to FACS tubes, the samples underwent three steps of washing with FACS buffer. A total of 10^4^ single-cell events were measured with the BD LSR II flow cytometer to obtain the mean fluorescence intensity (MFI).

The data were further analyzed and the cellular saturation *S* and the saturation half-time *t*_1/2_ were calculated using a coordinate transformation (*x* → −*x*; *y* → −*y*) and an exponential fit approximation that was iterated to reach a coefficient of variation of r^2^ = 1. This biophysical model considered an exponential convergence of the time-dependent uptake *I*(*t*) to a saturation limit according to Ashraf et al. (2020) [60], as shown in Equation (1):

(1) $$I\left(t\right)=S\left(1-exp\left(-\frac{t}{k}\right)\right)\;with\;k=t_{1/2}\ln\left(2\right)$$

#### 2.5.4. Cytotoxicity Assay

After seeding 2 × 10^4^ cells/well in 96-well plates, the cells were incubated for 48 h at 37 °C and 10% CO_2_. After 48 h, the medium was aspirated and replaced by 90% (90 µL) of the single well capacity (100 µL) with fresh medium. For the remaining 10% (10 µL) of the well capacity, the liposomal formulation dilutions were added. In accordance with the uptake tests, the liposomal formulations were added to achieve final lipid concentrations of 100, 500, and 1000 µM, and they were then incubated for 3 h. After adding 10 µL of alamarBlue™ HS (Thermo Fisher Scientific, Waltham, MA, USA) reagent and incubation for 2 h, the absorbance (wavelength 570 nm and reference wavelength 600 nm (Tecan Sunrise, Tecan Trading AG, Männedorf, Switzerland)) was measured on the multiplate reader to assess the vitality of the cells.

### 2.6. Statistical Analysis

Data are presented as means ± SD. To identify statistical differences between three or more different groups, two-way ANOVA with a post hoc test (Tukey’s multiple comparisons) was utilized. A *p*-value less than 0.05 was considered significant.

## 3. Results and Discussion

### 3.1. Characterization and Stability of Liposomes

The results of the characterization of size (Z-Average) and polydispersity index (PdI) of the liposomes are summarized in Table 2. All liposomal formulations showed a Z-Average (Z-Ave) of <130 nm and low PdI values < 0.2. In addition, all liposomes exhibited a monomodal size distribution. 

**Figure 2:**
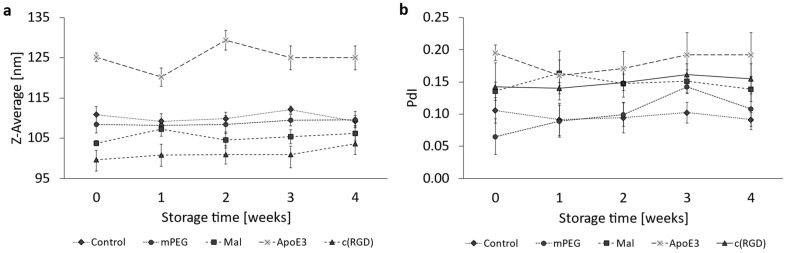
Liposomal stability over a storage period of 4 weeks at 4 °C. Representation of (**a**) the Z-Average and (**b**) the PdI. The bars represent the mean ± SD, n = 3.

The stability of the liposomal formulation was investigated over four weeks of storage at 4 °C under light-protected conditions. The results in Figure 2a show no significant changes in the liposomal size over time. The polydispersity index served as a further characteristic for assessing liposomal stability. As shown in Figure 2b, the fluctuations in the polydispersity index did not show a significant trend for all formulations over a period of 4 weeks. Linear regressions of the size and PdI evolution of the four liposomal formulations were analyzed by Student’s *t*-test. The individual graphs of the trend analysis for the Z-Ave (Supplementary Figure S1) and the PdI (Supplementary Figure S2), as well as a summarizing data table (Supplementary Table S1), can be found in the Supplementary Materials.

To estimate the conjugation efficiency more precisely, the apolipoprotein E3 concentration on the liposomal surface was determined using the Human ApoE ELISA ^BASIC^ Kit. Based on the determined concentration of 2.39 ± 0.38 µg/mL, the molar amount of ApoE3 *n_ApoE3_* was calculated and correlated with half of the molar amount of DSPE-PEG5k-Mal *n_Mal_*, as the distribution of this lipid anchor is assumed to be spread evenly on the inner and outer bilayer leaflet for symmetrical liposomes. The amount of DSPE-PEG5k-Mal *n_Mal_* was calculated via the cholesterol concentration of the final sterile filtrated liposomes. Since *n_ApoE3_*/*n_Mal_* = 0.129, this result documented a conjugation efficiency of 12.9%. The increase of the liposomal size of the liposomes by more than 20 nm (Mal liposomes have a Z-Ave of 103.8 ± 0.7 nm while ApoE3 liposomes 125.1 ± 1.1 nm) can be seen as additional confirmation of the successful conjugation of ApoE3 to the liposomal surface.

The determination of the thiol groups directly after the reaction of the protein with Traut’s reagent and after the final sterile filtration of the liposomes showed a reduction in the thiol groups by almost 90%. Conversely, this corresponds to a percentage of 10% remaining thiol groups. This indicates two aspects: (1) a successful conjugation of ApoE3 to the liposomal surface and an effective purification of the liposomes by dialysis, and (2) free thiol groups on already covalently bound ApoE3 on the liposomal surface, since the binding of ApoE3 to the liposome presumably occurs via several thiol groups. Therefore, the approach for thiolation and subsequent conjugation should be examined in more detail. The ApoE3 sequence contains a total of 13 lysines, of which most likely only 4 are relevant for thiolation using Traut’s reagent, due to their localization on the outside of the protein.

In addition, the maximum number of ApoE3 molecules per liposome was estimated by forming the ratio of the surface area of a spherical liposome *A_Liposome_* and the total cylindrical protein surface area *A_ApoE3_*. The result revealed that a maximum of nine ApoE3 molecules could be bound on the liposomal surface. This theoretical value was compared to the measured value of ApoE3 concentration as determined by ELISA. To do so, the number of ApoE3 molecules per milliliter *N_ApoE3_* was related to the number of liposomes per unit volume *N_Liposomes_* from the measured total lipid concentration. As a result, it was found that ~1 ApoE3 molecule was bound per liposome. 

### 3.2. Expression Levels of LRP1 and Integrin α_V_ in Endothelial and Cancer Cells

**Figure 3:**
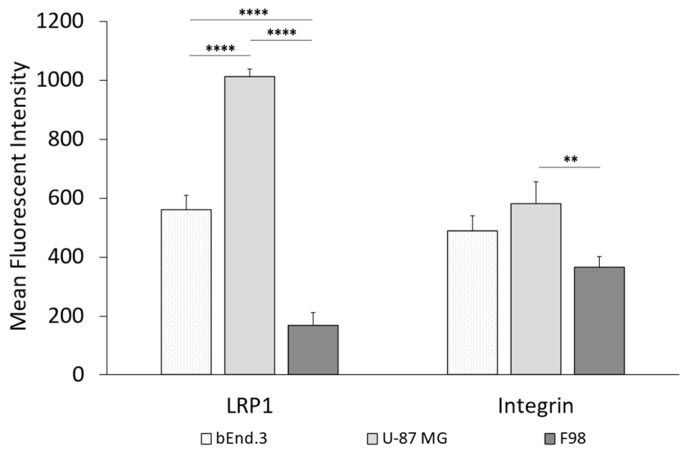
Expression levels of LRP1 and integrin receptor in cancer cells (U-87 MG and F98) and endothelial cells (bEnd.3). The bars represent the mean ± SD. Statistical analysis: one-way ANOVA followed by Tukey’s multiple comparison test. ** *p* < 0.01, **** *p* < 0.0001; n = 3.

Figure 3 presents the expression levels of the low-density lipoprotein receptor-related protein 1 (LRP1) and the integrin α_V_ receptor in the endothelial cell line bEnd.3, as well as in the two glioma cell lines U-87 MG and F98. The corresponding data table can be found in the Supplementary Materials (Table S2). The determination was carried out as described in Section 2.5.2.

As can be seen in Figure 3, the expression of both receptors was highest for the U-87 MG glioma cells. In the case of the LRP1 receptor, the expression was significant in U-87 MG cells compared to the rat glioma cell line F98 or the endothelial cells bEnd.3. Considering the integrin α_V_ receptor, there was only a significantly higher expression for the U-87 MG cells when comparing both tumor cell lines. No significant difference between the human glioma cells (U-87 MG) and the endothelial cells bEnd.3 could be detected.

Concerning LRP1, it is reported to be highly expressed in neurons [17], and its localization on the abluminal endothelial cell membrane [18] makes it an advantageous target. Furthermore, LRP1 is associated with the pathobiology of glioblastoma, as indicated by an increased expression in neoplastic glioblastoma cells [61,62,63,64]. Maletinska et al. (2000) were able to show that the LRP1 receptor is differentially expressed in seven different human glial cells, with expression in the U-87 MG cells being among the three highest [63]. Our results presented in Figure 3 are in line with Maletinska et al. 2000 [63], as LRP1 is expressed twice as highly in U-87 MG cells as compared to bEnd.3 cells. When comparing the two different glioma cell lines U-87 MG and F98, the LRP1 expression is increased by nearly six times in the human glioma cell line U-87 MG as compared to the rat F98 cells, as shown in Figure 3. 

On the other hand, so-called cell-penetrating peptides (CPPs), such as the cyclic RGD as a ligand for the integrin α_V_ receptor, have attracted more and more attention over the past decades. The first CPP was identified more than 25 years ago [65]. The integrin α_V_β_3_ receptor is overexpressed in tumor and angiogenic endothelial cells [66] and is correlated with poorer glioblastoma prognosis [67], which is in accordance with our results presented in Figure 3. There was no significant difference in the expression of the receptor between the glioma U-87 MG cells and the endothelial bEnd.3 cells, but when comparing the expression between both tumoral cell lines, a significant difference was found. The integrin α_V_ receptor was expressed at 6-fold higher rate in human U-87 MG cells than in rat F98 cells. 

### 3.3. Cellular Uptake of ApoE3-Modified Liposomes

**Figure 4:**
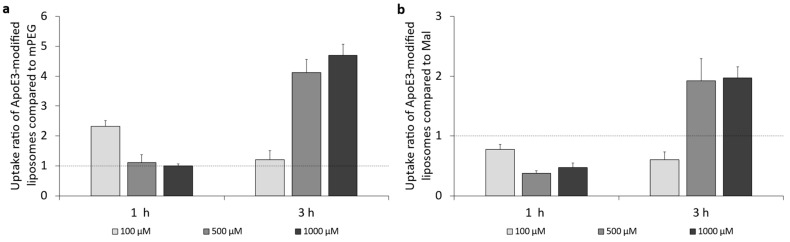
Uptake ratio of ApoE3-modified liposomes in bEnd.3 cells. (**a**) Liposomal uptake ratio of ApoE3-modified liposomes compared to mPEG liposomes in bEnd.3 cells after 1 h and 3 h of incubation, and (**b**) liposomal uptake factor of ApoE3-modified liposomes compared to Mal liposomes in bEnd.3 cells after 1 h and 3 h of incubation; n = 3.

The cellular uptake of liposomes modified with apolipoprotein E3 in bEnd.3 endothelial cells was studied according to Section 2.5.3. Uptake quantified by MFI of the ApoE3-modified liposomes was either compared to the MFI of PEGylated liposomes (Figure 4a) or the MFI of maleimide-functionalized liposomes (Figure 4b).

It was found that ApoE3-modification of the liposomal surface increased the cellular uptake, compared to PEGylated liposomes (Figure 4a), up to 2.3-fold when cells were incubated with 100 µM for 1 h. Increasing lipid concentration resulted in a decrease in uptake of ApoE3-modified liposomes, while an increase in incubation time to 3 h led to an increase in liposomal uptake up to 4.7-fold.

When comparing the uptake of ApoE3-modified liposomes to Mal (precursor) liposomes, there was no increased uptake at 1 h of incubation, as the ratio remained below 1.0. Exposure to medium and high lipid concentrations (500 µM and 1000 µM) at 3 h of incubation resulted in a 2-fold increase in cellular uptake of ApoE3-modified liposomes. 

**Figure 5:**
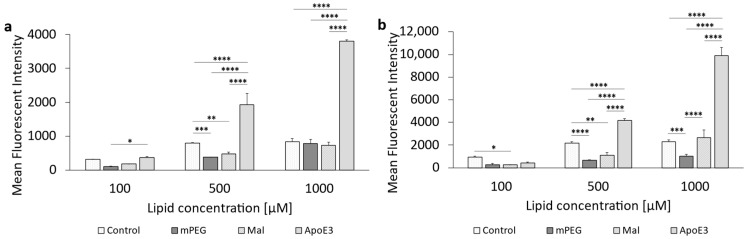
Uptake of ApoE3-modified liposomes in U-87 MG cells. (**a**) Liposomal uptake in U-87 MG cells after 1 h of incubation and (**b**) 3 h of incubation. The bars represent the mean ± SD. Statistical analysis: two-way ANOVA followed by a Tukey’s multiple comparison test. * *p* < 0.05, ** *p* < 0.01, *** *p* < 0.001, **** *p* < 0.0001; n = 3.

The following Figure 5 (cf. corresponding data in Table S4 in the Supplementary Materials) shows the concentration- and time-dependent uptake of the four liposomal formulations in the human glioblastoma cell line U-87 MG. The investigation of cellular uptake after 1 h of incubation showed that ApoE3-modified liposomes were always taken up most efficiently, and this was consistently significant compared to PEGylated liposomes. There was no significant difference in cellular uptake between the control liposomes, PEGylated liposomes, and the maleimide anchor liposomes at 100 µM or 1000 µM.

An increase in incubation time from 1 h to 3 h (Figure 5b) led to a general increased uptake for all liposomal formulations, and a similar trend of steadily increasing cellular uptake with increasing concentration was observed for the ApoE3-modified liposomes. It was also shown once again that PEGylated liposomes were taken up more poorly; in the case of 500 µM and also 1000 µM, the uptake was significantly reduced compared to the control liposomes.

**Figure 6:**
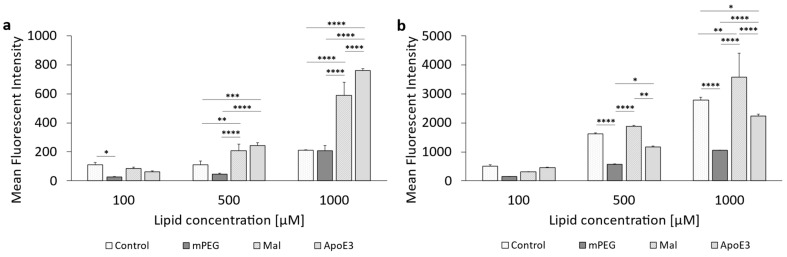
Uptake of ApoE3-modified liposomes in F98 cells. (**a**) Liposomal uptake in F98 after 1 h of incubation and (**b**) 3 h of incubation. The bars represent the mean ± SD. Statistical analysis: two-way ANOVA followed by a Tukey’s multiple comparison test. * *p* < 0.05, ** *p* < 0.01, *** *p* < 0.001, **** *p* < 0.0001; n = 3.

The rat-derived glioblastoma cell line F98 was used as a further tumor cell line to evaluate cellular uptake. The results are shown in Figure 6, with the corresponding data in Supplementary Table S5. It was shown that the ApoE3-modified liposomes were only taken up significantly higher at the highest concentration of 1000 µM after 1 h of incubation compared to the maleimide-anchor liposomes. A significantly higher uptake compared to PEGylated liposomes was shown at 500 µM.

In the case of the F98 cells, an extension of the incubation time (Figure 6b) did not lead to a comparable result to the 1 h of incubation. On the one hand, there was an almost linear increase in the cellular uptake of the control liposomes. Furthermore, a significant reduction in the cellular uptake of PEGylated liposomes was again observed above a tested liposomal concentration of 500 µM.

However, 3 h incubation showed that above a concentration of 500 µM, the maleimide-anchor liposomes were taken up at a significantly higher rate compared to the ApoE3-modified liposomes. 

When cellular uptake was compared with the expression of the receptor, a correlation can be found. This can be seen, for example, when comparing the uptake after 1 h (Figure 5a) at 100 µM. In this case, the uptake of the ApoE-3-modified liposomes at 100 µM in U-87 MG cells was increased 6-fold compared to F98 cells. Even with increasing lipid concentrations to 500 µM or 1000 µM, the uptake in the U-87 MG cells remained roughly 6-fold higher compared to F98 cells.

**Figure 7:**
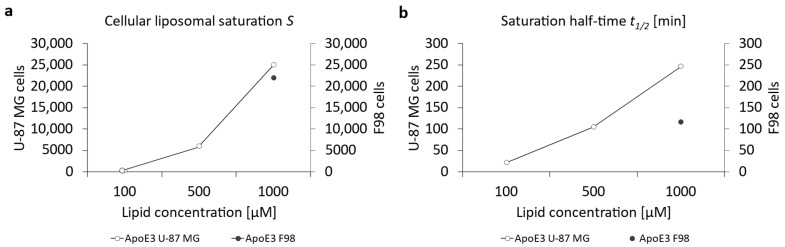
Calculation of (**a**) the cellular liposomal saturation *S* and (**b**) the saturation half-time *t*_1/2_ vs. lipid concentration of ApoE3-modified liposomes in U-87 MG and F98 cells according to Equation (1). In those cases where the exponential regression was only possible with a coefficient of variation r^2^ < 1, the data points were discarded due to insufficient accuracy.

For a more precise comparison between those two cell lines, Figure 7a represents the cellular liposomal saturation *S* and the saturation half-time *t*_1/2_ (Figure 7b). Table S10 summarizes the data for the cellular liposomal saturations *S* and saturation half-time *t*_1/2_. Figure S7a contains, exemplarily, the cellular liposomal saturation S and the cellular saturation half-time *t*_1/2_ as red lines. Supplementary graphs for the ApoE3-modified liposomes are included in Figure S6.

There were almost identical saturation values (Figure 7a) for both cell lines of ~25,000 for U-87 MG cells and ~22,000 for F98 cells, but enormous differences are observed if the saturation half-time is considered as an indicator of the “speed of uptake” in Figure 7b. Uptake of the ApoE3-modified liposomes occurred in U-87 cells at more than twice the speed of F98 cells. This contrast of very similar saturations despite large differences in uptake speed measured at 1 h or 3 h can be explained by a cell biological effect: continuous endocytosis via the LRP1 receptor leads to a depletion of LRP1 at the plasma membrane. However, LRP1 is intracellularly recycled through different pathways and starts to re-appear on the plasma membrane surface around 90 min after internalization [68,69,70]. Additionally, the de novo biosynthesis takes more than 4 h, and therefore also contributed to the continuous uptake of ApoE3-modified liposomes via the LRP1 receptor.

In general, our results are in accordance with what has been demonstrated by others: the ApoE3-modified formulation showed an increased uptake in endothelial cells or higher penetration in co-culture models [20,28,71,72].

### 3.4. Cellular Uptake of c(RGD)-Modified Liposomes

**Figure 8:**
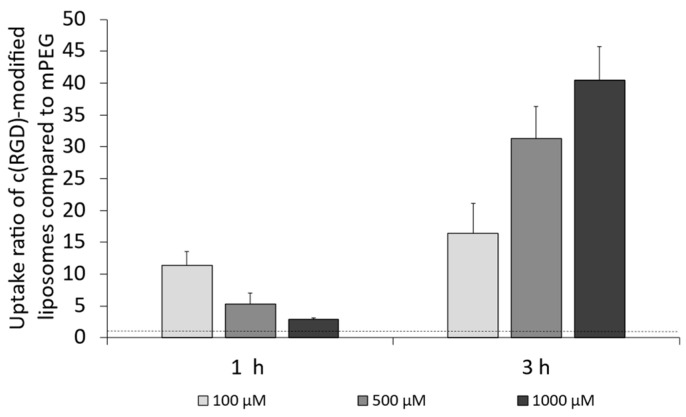
Uptake ratio of c(RGD)-modified liposomes in bEnd.3 cells compared to mPEG liposomes in bEnd.3 cells after 1 h and 3 h of incubation; n = 3.

In addition to apolipoprotein E3 as a possible ligand for the LRP1 receptor, the cyclic tripeptide c(RGD) on the liposomal surface was investigated as a ligand for the integrin α_V_ receptor. The experiments to determine the cellular uptake in the endothelial cell line bEnd.3 as well as the two tumoral cell lines U-87 MG and F98 were also carried out according to Section 2.5.3. Figure 8 summarizes the results after 1 h and 3 h of incubation of the bEnd.3 cells with the three different liposomal formulations. The corresponding data can be found in the Supplementary Table S3. 

In general, it was found that c(RGD)-modification of the liposomal surface resulted in an enormous increase in cellular uptake, as the uptake ratio when comparing the uptake with PEGylated liposomes was above 1.0 in all cases (concentrations and duration of incubation). 

Considering the results obtained after 1 h of incubation, there was a decrease in the uptake ratio of c(RGD)-modified liposomes when compared to mPEG liposomes for an increase in the tested lipid concentration. Contrary to this, there was an increase in the uptake ratio with increasing lipid concentration for 3 h of incubation. A 3 h incubation of the bEnd.3 cells (Figure 8) showed from a 16.4-fold up to a 40.5-fold increase with increasing concentration. 

**Figure 9:**
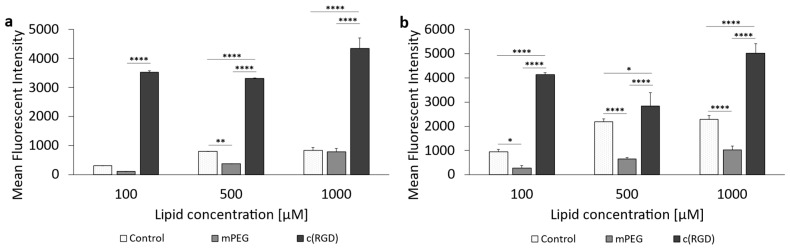
Uptake of c(RGD)-modified liposomes in U-87 MG cells. (**a**) Liposomal uptake in U-87 MG cells after 1 h of incubation and (**b**) 3 h of incubation. The bars represent the mean values with the standard deviation as error bars. Statistical analysis: two-way ANOVA followed by a Tukey’s multiple comparison test. * *p* < 0.05, ** *p* < 0.01, **** *p* < 0.0001; n = 3.

Figure 9 presents the results of liposomal uptake in U-87 MG cells (cf. corresponding data in Table S4 in the Supplementary Materials). Except for the 500 µM concentration, there was no significant difference between the uptake of the control liposomes and the PEGylated liposomes when incubated for 1 h (Figure 9a). In comparison, there were significant differences between the cellular uptake of the control liposomes compared to the PEGylated liposomes when incubated for 3 h at all concentrations (Figure 9b). The c(RGD) modified liposomes had the significantly highest cellular uptake at all three concentrations, both at 1 h and 3 h incubation. However, there was no concentration-dependent increase in uptake.

**Figure 10:**
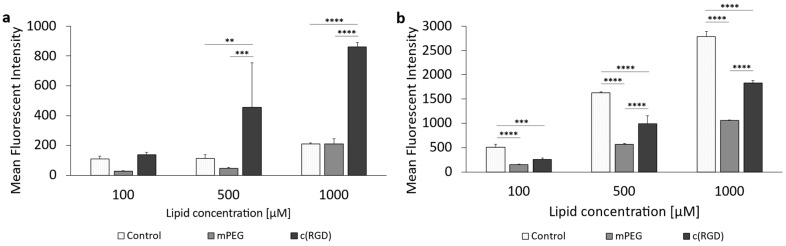
Uptake of c(RGD)-modified liposomes in F98 cells. (**a**) Liposomal uptake in F98 cells after 1 h of incubation and (**b**) 3 h of incubation. The bars represent the mean values with the standard deviation as error bars. Statistical analysis: two-way ANOVA followed by a Tukey’s multiple comparison test, ** *p* < 0.01, *** *p* < 0.001, **** *p* < 0.0001; n = 3.

The cellular uptake of c(RGD) modified liposomes was also assessed in rat F98 cells. The results are given in Figure 10. The corresponding data can be found in the Supplementary Table S5. For the c(RGD) liposomes, 1 h incubation (Figure 10a) showed an almost linear increase in cellular uptake with increasing concentration. In the case of 500 µM and also 1000 µM, the uptake of the c(RGD) modified liposomes was significantly higher than the uptake of the control liposomes and mPEG liposomes. The uptake of the control liposomes and the PEGylated liposomes did not differ significantly at any of the concentrations tested during a 1 h incubation.

In contrast to the 1 h incubation, all three liposomal formulations showed a concentration-dependent increase in cellular uptake with prolonged incubation time (Figure 10b). The control liposomes were taken up at the highest rate at all three liposomal concentrations tested, and this result was significant.

In the case of the c(RGD)-modified liposomes, no comparatively precise correlation was found between the expression of the target and the cellular uptake of the liposomes as with the ApoE3-modified liposomes, since the uptake behavior of c(RGD)-modified liposomes in both tumoral cell lines was fundamentally different. The F98 cells showed both time- and concentration-dependent uptake, whereas the U-87 cells appeared to have reached a kind of “maximum” even at the lowest lipid concentration and incubation over 1 h; uptake could not be increased either by increasing lipid concentration or by increasing the incubation time. One possible reason for this could be the comparatively high cytotoxicity of the formulation on the U-87 MG cells, as the relative viability was reduced to as low as 51.7% at the highest lipid concentrations of 1000 µM. This might be due to the fact that c(RGD) occupies the integrin receptors on the cell surface and inhibits integrin-mediated cell adhesion, leading to cell detachment and even apoptosis [73,74].

**Figure 11:**
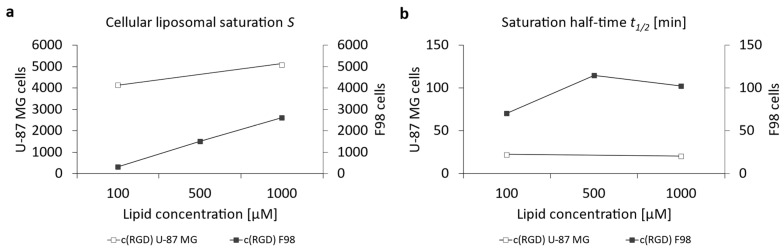
Representation of the (**a**) cellular liposomal saturation *S* and (**b**) saturation half-time *t*_1/2_ vs. lipid concentration of c(RGD)-modified liposomes in U-87 MG and F98 cells as calculated according to Equation (1). In those cases where the exponential regression only produced r^2^ < 1, the data points were discarded due to insufficient accuracy.

A closer look at the results of the uptake can also be obtained using the cellular saturation (Figure 11a) and the saturation half-time (Figure 11b). Table S10 summarizes the data for the cellular liposomal saturations *S* and saturation half-time *t*_1/2_. Supplementary graphs for c(RGD)-modified liposomes are included in Figure S7. The saturation values for c(RGD)-modified liposomes in U-87 MG cells was 1.95-fold higher compared to F98 cells, whereas the uptake rate in the F98 cells was 5-fold higher compared to the U-87 cells. Chen et al. (2012) showed a 2.5-fold increase in the uptake of RGD-modified liposomes when compared to PEGylated liposomes [75]. Dou et al. (2018) showed that the cellular uptake of c(RGD)-modified liposomes was correlated with receptor expression via comparison of the uptake in high-expression human glioma U-87 MG cells and low-expression human myelogenous leukemia K562 cells [76].

The cytotoxicity of all liposomal formulations across the three concentrations, 100 µM, 500 µM, and 1000 µM, was determined for all three cell lines in order to be able to assess the basic toxicity of the liposomes without an encapsulated active substance. The results can be found in Supplementary Figures S3–S5, with corresponding data in Tables S6–S8. In the case of bEnd.3 cells, comparable cell viabilities were observed for all liposomal formulations at 1000 µM, except for c(RGD)-modified liposomes, where cell viability was reduced to below 50%. In the case of U-87 MG glioma cells, all formulations exerted a higher cytotoxicity, whereby this was most pronounced with mPEG liposomes. Cell viability was reduced to almost 40% at 1000 µM. For the F98 cells, the results were comparable to the bEnd.3 cells, as here too the c(RGD)-modified liposomes caused the highest reduction in cell viability. In general, ApoE3-modified liposomes showed the lowest cytotoxicity for all tested cell lines. This could be due to an increased ATP concentration and thus increased energy potential in the cells due to the presence of ApoE3, as already shown by [77] in mice and transgenic cells.

## 4. Conclusions

In this study, a targeted drug delivery system with either ApoE3 or c(RGD) on the surface was successfully constructed for glioblastoma therapy and produced promising in vitro data regarding the possibility of elevated cellular uptake.

Our results indicate that the selection of a ligand is less dependent on its chemical species (peptide or protein) than on the target structure (cell type). Thus, the ApoE3-modification of liposomes is a favorable strategy for tumor cell targeting but it might be also a good ligand candidate for BBB passaging, as the uptake is increased in endothelial cells at higher lipid concentrations. As these high lipid concentrations are non-therapeutic in vivo, it will be of interest to increase the ApoE3-surface density on the liposomes for an increased uptake even at lower lipid concentrations. Contrarily, liposomes modified with the cyclic RGD peptide showed a stronger increase in uptake in endothelial cells.

In most cases, surface modifications with either ApoE3 or c(RGD) showed a time- and concentration-dependent manner of uptake for all cell lines, except for U-87 MG cells. In the case of the human glioma cells, a saturation of uptake could be seen. To answer the question of whether a correlation of liposomal cellular uptake with the expression of the receptor can be established, an affirmative response is only possible for ApoE3-modified liposomes.

Regarding the uptake of either ApoE3- or c(RGD)-modified liposomes, there were enormous differences in cellular uptake between the two different glioma cell lines of different origin. However, it is important to add that the uptake of the ApoE3-modified liposomes resulted in a comparable saturation of F98 cells and U-87 MG cells despite lower expression of LRP1 in F98 cells and a significantly slower uptake rate.

Our current studies do not allow an absolute statement as to whether the liposomes are completely transcytosed in the endothelial cells. Therefore, it is of interest to evaluate the BBB passage using a transwell model. Investigating the liposomal uptake in different glioma cells of the same species (e.g., patient-derived) as well as an investigation on the liposomal uptake in non-targeted cells can complement the data.

## Figures and Tables

**Table 1:** Composition of liposomal formulations.

Sample Nomenclature	Control	mPEG	Mal	ApoE3	c(RGD)
Components	Molar Ratio [%]				
DPPC	59.9	54.9	54.8	54.8	49.9
Cholesterol	40	40	40	40	40
DSPE-PEG2k	-	5	5	5	5
DSPE-PEG5k-Mal	-	-	0.1	-	-
DSPE-PEG5k-Mal-ApoE3	-	-	-	0.1	-
DSPE-PEG5k-c(RGDyk)	-	-	-	-	5
DiD	0.1	0.1	0.1	0.1	0.1

**Table 2:** Composition, particle size (Z-Average), and polydispersity index of the tested liposomal formulations.

Liposomes	Z-Average [nm]	PdI
Control	110.8 ± 2.0	0.106 ± 0.020
mPEG	108.5 ± 1.9	0.065 ± 0.028
Mal	103.8 ± 0.7	0.136 ± 0.014
ApoE3	125.1 ± 1.1	0.195 ± 0.012
c(RGD)	99.6 ± 2.3	0.143 ± 0.037

## Data Availability

The data supporting this article have been included as part of the Supplementary Information.

## Supplementary Materials

The following supporting information can be downloaded at: https://www.mdpi.com/article/10.3390/pharmaceutics16091112/s1, Figure S1: Trend analysis of the Z-Average of the liposomal formulations over a storage period of 4 weeks at 4 °C. Determination of significance of a linear regression using Student’s *t*-test. Figure S2: Trend analysis of the PdI of the liposomal formulations over a storage period of 4 weeks at 4 °C. Determination of significance of a linear regression using Student’s *t*-test. Figure S3: Liposomal cytotoxicity on bEnd.3 cells using alamarBlue™ HS reagent. Representation of all different tested formulations (a) to (f). The bars represent the mean values with the standard deviation as error bars. Statistical analysis: one-way ANOVA followed by Tukey’s multiple comparison test. * *p* < 0.05, ** *p* < 0.01, *** *p* < 0.001; n = 3. Figure S4: Liposomal cytotoxicity on U-87 MG cells using alamarBlue™ HS reagent. Representation of all different tested formulations (a) to (f). The bars represent the mean values with the standard deviation as error bars. Statistical analysis: one-way ANOVA followed by Tukey’s multiple comparison test. * *p* < 0.05, ** *p* < 0.01, *** *p* < 0.001, **** *p* < 0.0001; n = 3. Figure S5: Liposomal cytotoxicity on F98 cells using alamarBlue™ HS reagent. Representation of all different tested formulations (a) to (f). The bars represent the mean values with the standard deviation as error bars. Statistical analysis: one-way ANOVA followed by Tukey’s multiple comparison test. * *p* < 0.05, ** *p* < 0.01, *** *p* < 0.001, **** *p* < 0.0001; n = 3. Figure S6: Plot of the mean fluorescence intensities over time (a and c) and the corresponding exponential fits after a transformation of the coordinates (x → −x; y → −y) (b,d) for the cellular uptake of ApoE3-modified liposomes into the different glioblastoma cells, where. (a,b) represent the data for the U-87 MG cells and, (c,d) for the F98 cells. Figure S7: Plot of the mean fluorescence intensities over time (a,c) and the corresponding exponential fits (b,d) for the cellular uptake of c(RGD)-modified liposomes into the different glioblastoma cells, where. (a,b) represent the data for the U-87 MG cells and, (c,d) for the F98 cells. Table S1: Overview of particle size (Z-Average) and polydispersity index (PdI) over a storage period of 4 weeks at 4 °C. Values are given as mean ± SD, n = 3. Table S2: Mean fluorescent intensities (MFI) for the LRP1 and Integrin αV staining at different glucose concentrations in bEnd.3 cells, U-87 MG cells and F98 cells, n = 3. Table S3: Uptake ratio of ApoE3- or c(RGD)-modified liposomes compared to different reference liposomes (mPEG or Mal) in bEnd.3 cells after 1 h and 3 h of incubation, n = 3. Table S4: Mean fluorescent intensities (MFI) for the liposomal uptake in U-87 MG glioma cells, n = 3. Table S5: Mean fluorescent intensities (MFI) for the liposomal uptake in F98 glioma cells, n = 3. Table S6: Values of the relative viability of the bEnd.3 cells, n = 3. Table S7: Values of the relative viability of the U-87 MG cells, n = 3. Table S8: Values of the relative viability of the F98 cells, n = 3. Table S9 Calculated cellular liposomal saturation (S) and the corresponding saturation half-time (*t*_1/2_) for U-87 MG cells and F98 cells.
