# Supplementary material for: Increased Cellular Uptake of ApoE3- or c(RGD)-Modified Liposomes for Glioblastoma Therapy Depending on the Target Cells
Source: Pharmaceutics. 2024 Aug 23;16(9):1112. doi: 10.3390/pharmaceutics16091112 (PMC11434700; doi:10.3390/pharmaceutics16091112)
Supplement: Supplementary file 1 [file pharmaceutics-16-01112-s001.zip › pharmaceutics-3145245-supplementary.pdf]

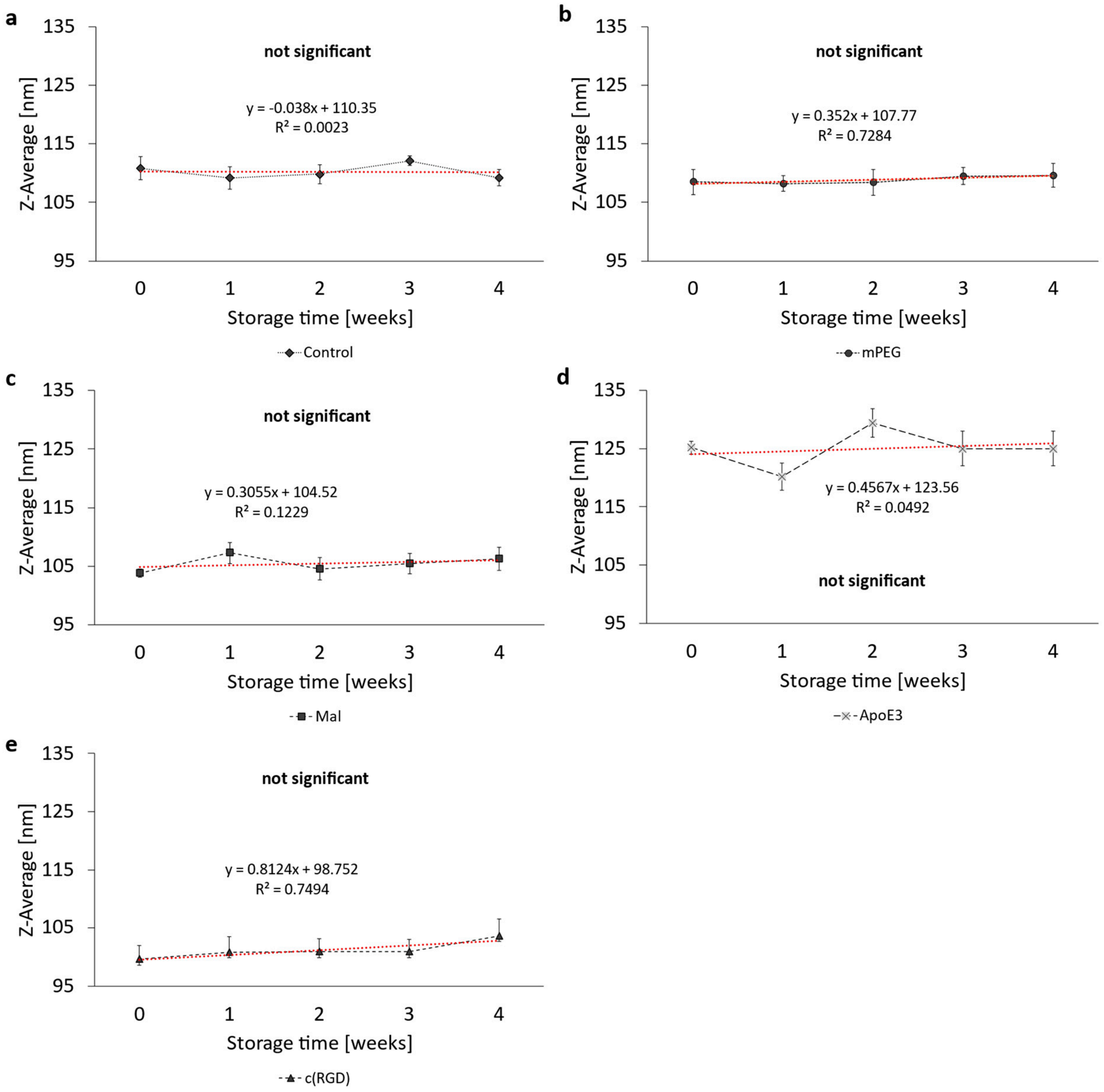

Figure S1: Trend analysis of the Z-Average of the liposomal formulations over a storage period of 4 weeks at 4 °C. Determination of significance of a linear regression using Student's t-test.

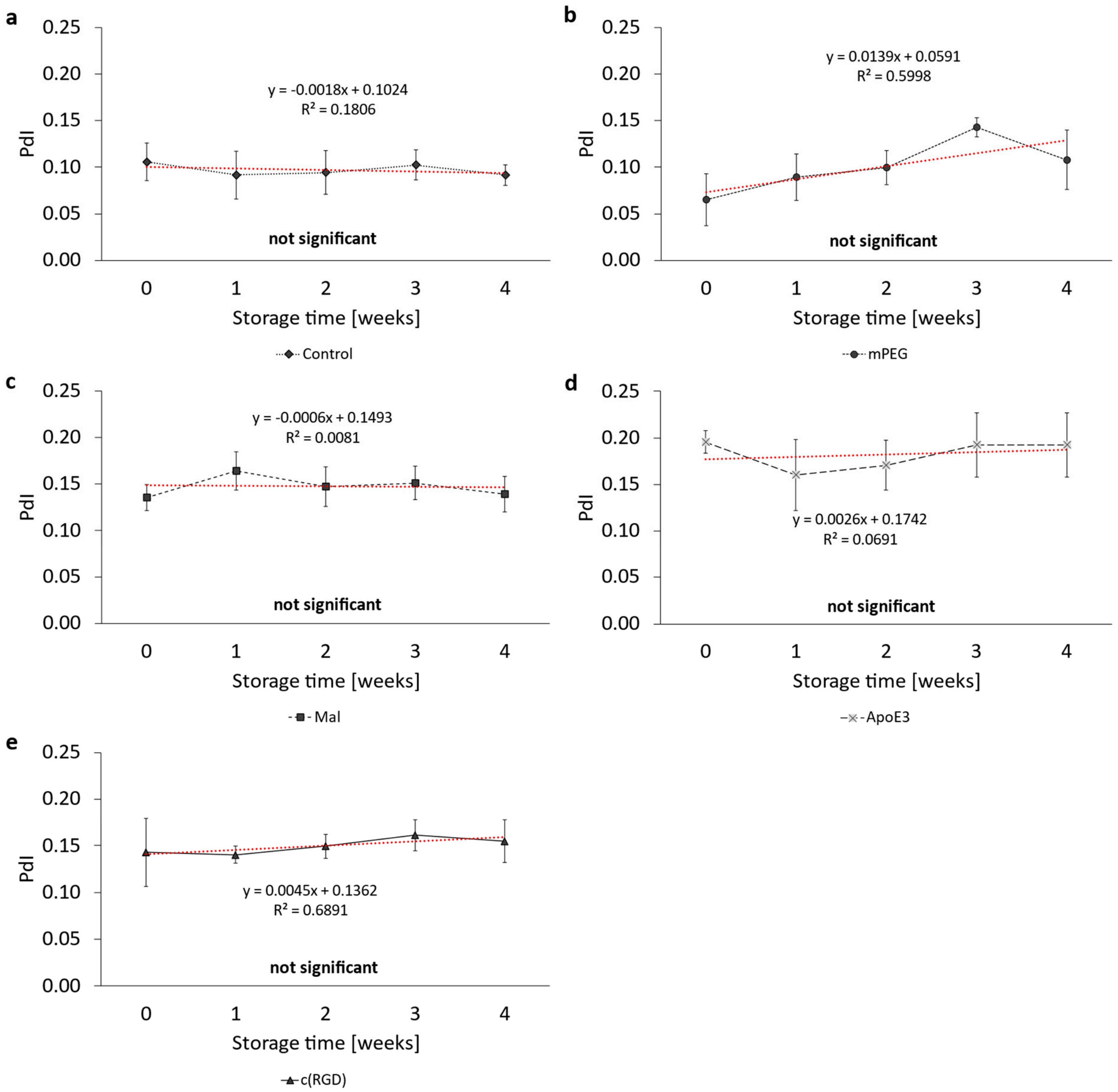

Figure S2: Trend analysis of the PdI of the liposomal formulations over a storage period of 4 weeks at 4 °C. Determination of significance of a linear regression using Student's t-test.

Table S1: Overview of particle size (Z-Average) and polydispersity index (PdI) over a storage period of 4 weeks at 4 °C. Values are given as mean ± SD, n = 3.

Type of liposome	Storage time [weeks]	Z-Average [nm]		PdI	
		Mean	SD	Mean	SD
**Control**	0	110.8	2.0	0.106	0.020
	1	109.2	1.9	0.092	0.025
	2	109.8	1.6	0.094	0.023
	3	112.1	0.8	0.102	0.016
	4	109.2	1.4	0.092	0.011
**mPEG**	0	108.5	2.1	0.065	0.028
	1	108.2	1.3	0.089	0.025
	2	108.4	2.2	0.100	0.018
	3	109.5	1.4	0.143	0.011
	4	109.6	2.0	0.108	0.032
**Mal**	0	103.8	0.7	0.136	0.014
	1	107.3	1.8	0.164	0.020
	2	104.5	1.9	0.147	0.021
	3	105.4	1.7	0.151	0.018
	4	106.2	1.9	0.139	0.019
**ApoE3**	0	125.1	1.1	0.195	0.012
	1	120.2	2.3	0.160	0.038
	2	129.4	2.5	0.171	0.027
	3	125.0	3.0	0.192	0.034
	4	125.0	3.0	0.192	0.034
**c(RGD)**	0	99.6	2.3	0.143	0.037
	1	100.8	2.6	0.140	0.009
	2	100.9	2.2	0.149	0.013
	3	100.9	2.1	0.161	0.017
	4	103.7	2.8	0.155	0.023

Table S2: Mean fluorescent intensities (MFI) for the LRP1 and Integrin αv staining at different glucose concentrations in bEnd.3 cells, U-87 MG cells and F98 cells, n = 3.

Receptor	bEnd.3 cells		U-87 MG cells		F98	
	Mean	SD	Mean	SD	Mean	SD
LRP1	560.0	48.2	1012.3	25.6	169.0	42.3
Integrin αv	487.5	53.0	580.5	74.7	365.8	36.5

Table S3: Uptake ratio of ApoE3- or c(RGD)-modified liposomes compared to different reference liposomes (mPEG or Mal) in bEnd.3 cells after 1 h and 3 h of incubation, n = 3.

**Time of incubation**		**100 μM**		**500 μM**		**1000 μM**	
		**Mean**	**SD**	**Mean**	**SD**	**Mean**	**SD**
1 h	**ApoE3/mPEG**	2.3	0.2	1.1	0.3	1.0	0.1
	**ApoE3/Mal**	0.8	0.1	0.4	0.0	0.5	0.1
	**c(RGD)/mPEG**	11.4	2.1	5.3	1.7	2.9	0.2
3 h	**ApoE3/mPEG**	1.2	0.3	4.1	0.4	4.7	0.4
	**ApoE3/Mal**	0.6	0.1	1.9	0.4	2.0	0.2
	**c(RGD)/mPEG**	16.4	4.7	31.3	5.1	40.5	5.2

Table S4: Mean fluorescent intensities (MFI) for the liposomal uptake in U-87 MG glioma cells, n = 3.

**Type of liposome**	**Concentration [µM]**	**1 hour of incubation**		**3 hours of incubation**	
		**Mean**	**SD**	**Mean**	**SD**
**Control**	100	312.2	4.6	956.1	83.5
	500	798.9	13.8	2187.0	116.6
	1000	839.2	94.1	2283.0	154.2
**mPEG**	100	109.5	8.8	279.5	98.5
	500	378.2	7.7	656.3	52.9
	1000	789.0	114.9	1028.1	146.8
**Mal**	100	179.6	8.5	248.3	25.0
	500	480.6	49.5	1091.8	242.1
	1000	735.1	84.6	2657.3	666.3
**ApoE3**	100	370.3	24.1	431.7	78.3
	500	1934.0	332.4	4161.7	184.8
	1000	3803.0	43.9	9902.0	717.9
**c(RGD)**	100	3526.1	55.0	4131.9	74.8
	500	3323.9	15.3	2842.9	551.4
	1000	4345.7	359.1	5018.3	406.6

Table S5: Mean fluorescent intensities (MFI) for the liposomal uptake in F98 glioma cells, n = 3.

**Type of liposome**	**Concentration [µM]**	**1 hour of incubation**		**3 hours of incubation**	
		**Mean**	**SD**	**Mean**	**SD**
**Control**	100	110.62	16.7	509.8	53.1
	500	111.48	26.1	1628.5	23.5
	1000	210.78	4.5	2787.0	102.9
**mPEG**	100	28.01	2.0	149.2	10.9
	500	46.94	6.1	569.5	20.8
	1000	209.38	34.9	1058.4	8.4
**Mal**	100	85.55	10.4	318.7	1.9
	500	209.53	43.7	1884.5	30.5
	1000	589.14	91.9	3584.7	822.9
**ApoE3**	100	62.4	6.8	464.3	5.9
	500	243.3	19.4	1177.3	29.2
	1000	760.7	14.3	2239.7	66.2
**c(RGD)**	100	137.5	17.1	257.6	26.5
	500	453.9	299.2	994.9	156.8
	1000	860.6	27.4	1831.0	47.0

Table S6: Values of the relative viability of the bEnd.3 cells, n = 3.

Type of liposome	Concentration [µM]	Relative Viability [%]	
		Mean	SD
**Control**	100	94.68	3.01
	500	85.36	0.66
	1000	75.62	4.32
**mPEG**	100	99.24	4.07
	500	87.02	10.69
	1000	78.00	7.16
**Mal**	100	90.32	3.24
	500	75.08	4.65
	1000	77.03	6.60
**ApoE3**	100	93.99	5.14
	500	90.55	11.64
	1000	76.44	9.05
**c(RGD)**	100	80.30	5.51
	500	64.14	2.21
	1000	48.18	3.60

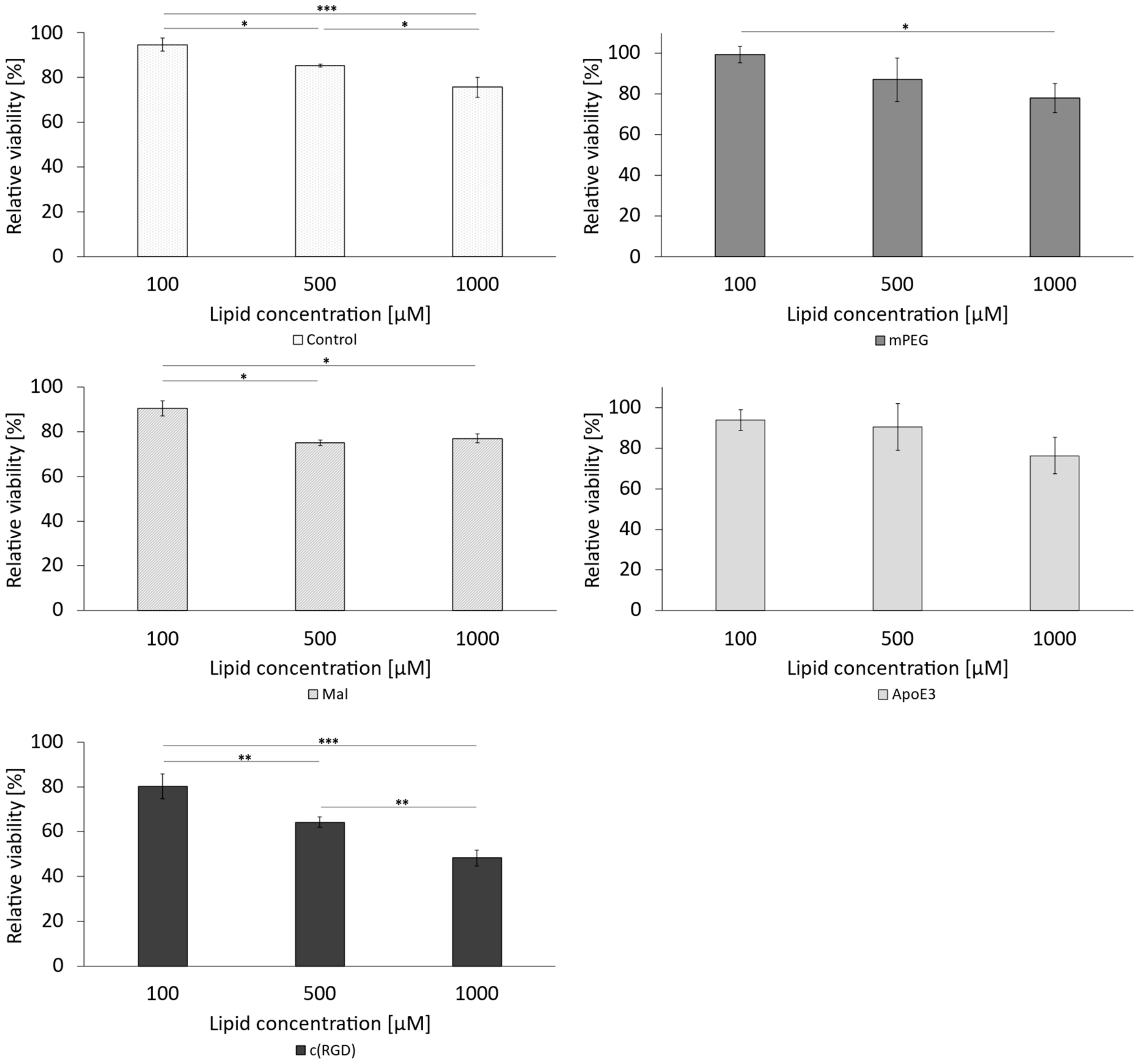

Figure S3: Liposomal cytotoxicity on bEnd.3 cells using alamarBlue™ HS reagent. Representation of all different tested formulations (a) to (f). The bars represent the mean values with the standard deviation as error bars. Statistical analysis: one-way ANOVA followed by Tukey's multiple comparison test. *$p < 0.05$, **$p < 0.01$, ***$p < 0.001$; n = 3.

Table S7: Values of the relative viability of the U-87 MG cells, n = 3.

Type of liposome	Concentration [µM]	Relative Viability [%]	
		Mean	SD
**Control**	100	94.71	12.12
	500	87.11	13.37
	1000	59.49	4.50
**mPEG**	100	99.38	5.45
	500	72.83	6.65
	1000	42.42	5.26
**Mal**	100	97.73	10.25
	500	82.53	9.24
	1000	56.98	10.59
**ApoE3**	100	97.21	2.35
	500	80.94	2.73
	1000	78.22	3.13
**c(RGD)**	100	102.39	9.97
	500	74.33	4.64
	1000	54.91	7.52

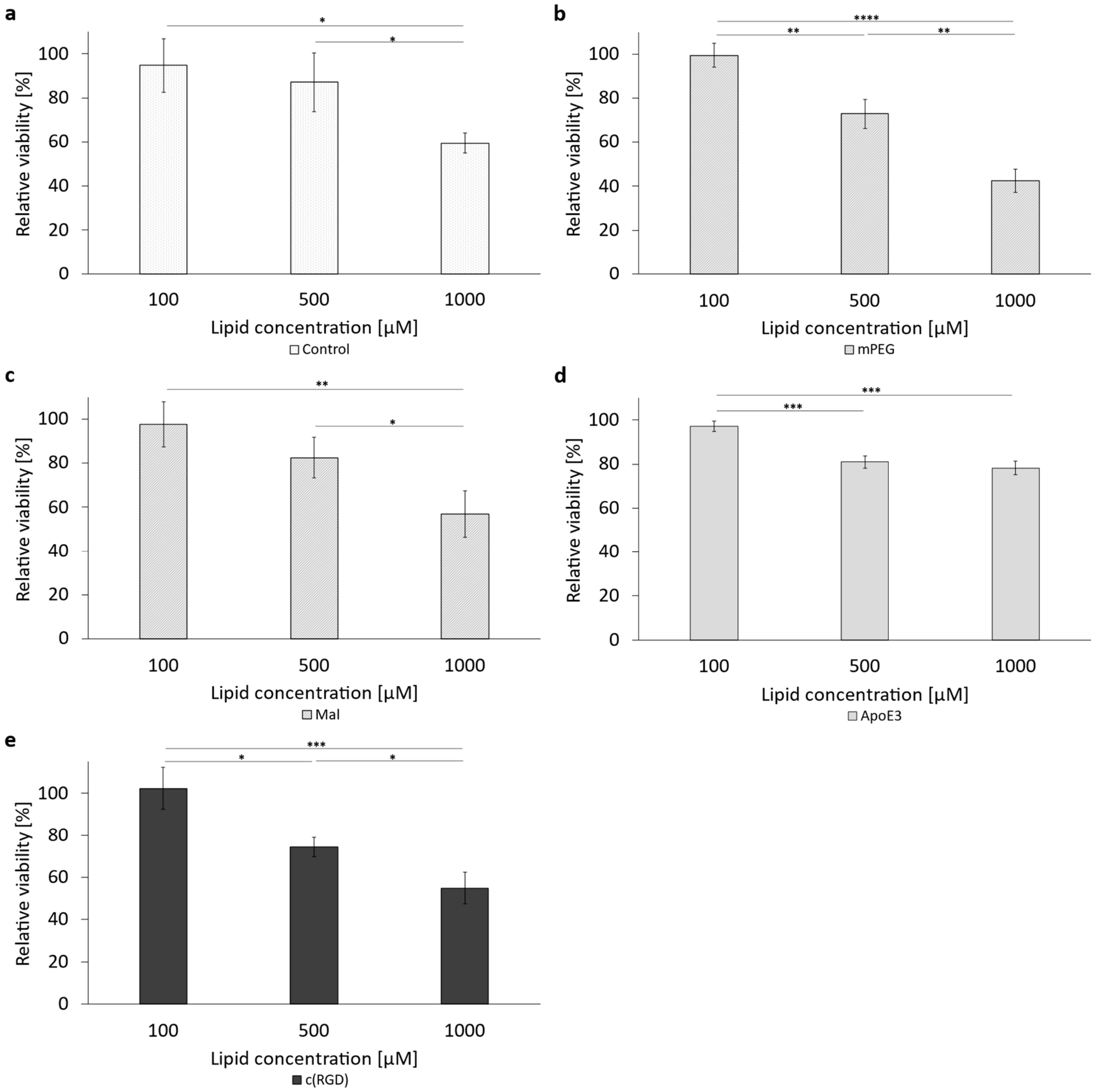

Figure S4: Liposomal cytotoxicity on U-87 MG cells using alamarBlue™ HS reagent. Representation of all different tested formulations (a) to (f). The bars represent the mean values with the standard deviation as error bars. Statistical analysis: one-way ANOVA followed by Tukey's multiple comparison test. *$p < 0.05$, **$p < 0.01$, ***$p < 0.001$, ****$p < 0.0001$; n = 3.

Table S8: Values of the relative viability of the F98 cells, n = 3.

Type of liposome	Concentration [µM]	Relative Viability [%]	
		Mean	SD
**Control**	100	96.82	1.00
	500	83.11	2.98
	1000	75.77	2.13
**mPEG**	100	97.15	2.93
	500	77.63	2.13
	1000	66.23	3.91
**Mal**	100	97.67	5.29
	500	84.38	7.88
	1000	74.31	5.00
**ApoE3**	100	99.08	3.01
	500	92.33	3.12
	1000	83.17	7.29
**c(RGD)**	100	87.90	4.35
	500	72.90	5.76
	1000	51.75	5.59

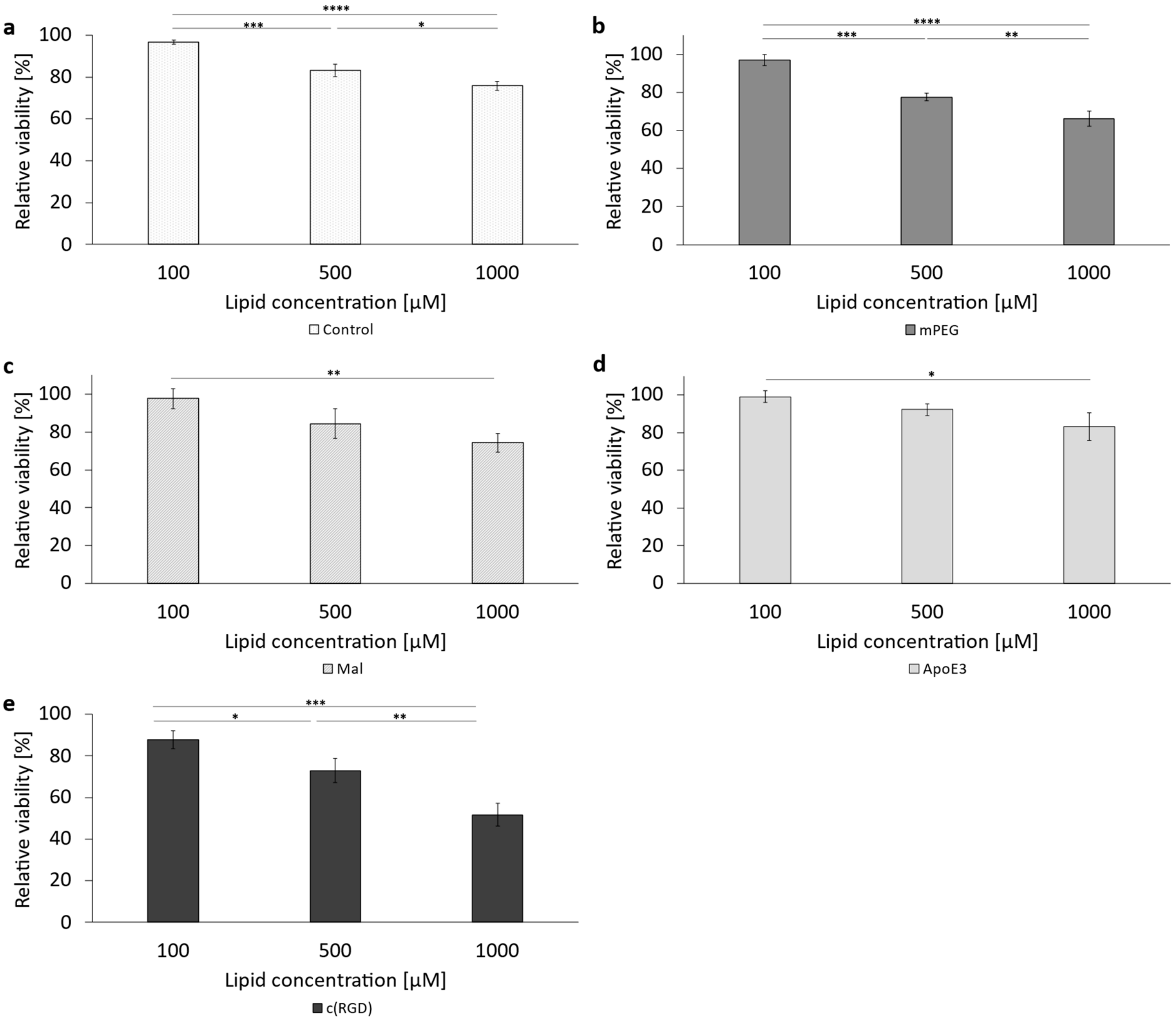

Figure S5: Liposomal cytotoxicity on F98 cells using alamarBlue™ HS reagent. Representation of all different tested formulations (a) to (f). The bars represent the mean values with the standard deviation as error bars. Statistical analysis: one-way ANOVA followed by Tukey's multiple comparison test. *$p < 0.05$, **$p < 0.01$, ***$p < 0.001$, ****$p < 0.0001$; n = 3.

Table S9: Calculated cellular liposomal saturation (*S*) and the corresponding saturation half-time ($t_{1/2}$) for U-87 MG cells and F98 cells.

		U-87 MG cells		F98 cells	
**Type of liposome**	**Concentration [µM]**	**Cellular liposomal saturation *S***	**Saturation half-time $t_{1/2}$ [min]**	**Cellular liposomal saturation *S***	**Saturation half-time $t_{1/2}$ [min]**
**ApoE3**	100	432.03	21.58	-	-
	500	6013.40	105.37	-	-
	1000	25033.00	247.11	22006.00	116.17
**c(RGD)**	100	4136.90	21.95	310.85	70.11
	500	-	-	1501.60	114.57
	1000	5086.90	20.56	2607.20	102.59

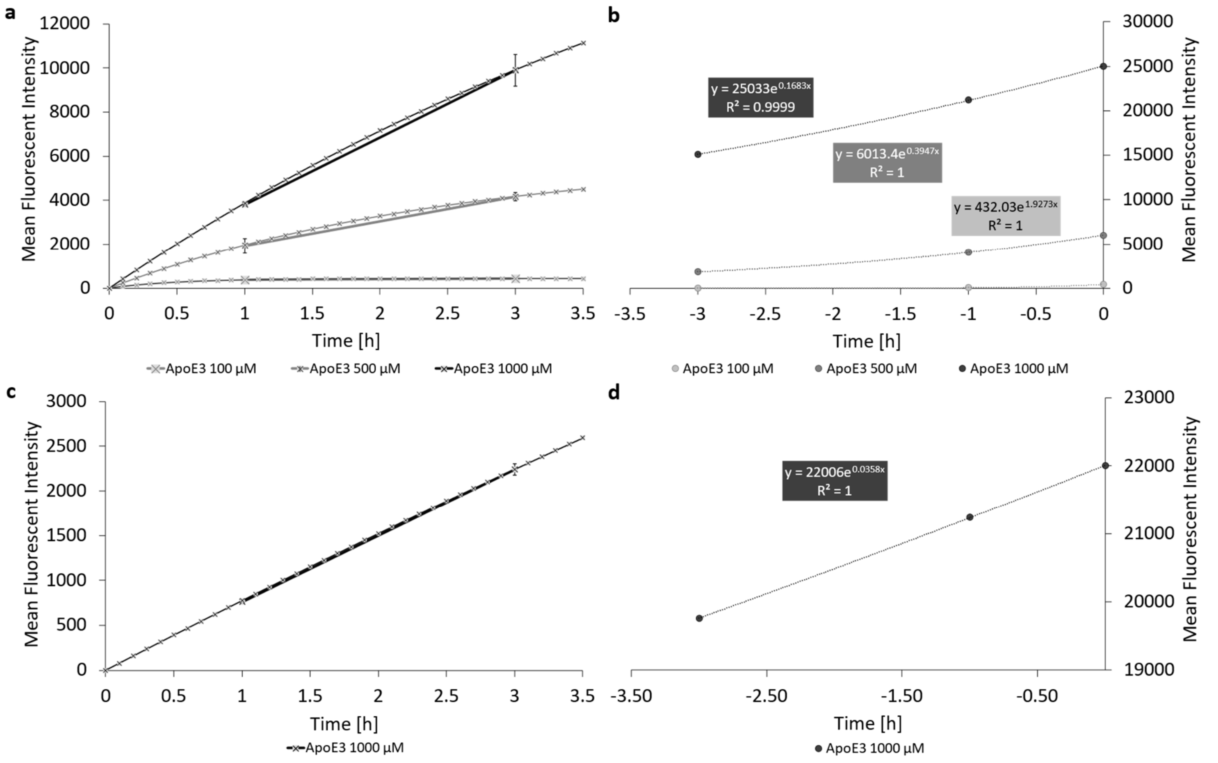

Figure S6: Plot of the mean fluorescence intensities over time (a and c) and the corresponding exponential fits after a transformation of the coordinates ($x \rightarrow -x; y \rightarrow -y$) (b and d) for the cellular uptake of ApoE3-modified liposomes into the different glioblastoma cells, where. (a and b) represent the data for the U-87 MG cells and, (c and d) for the F98 cells.

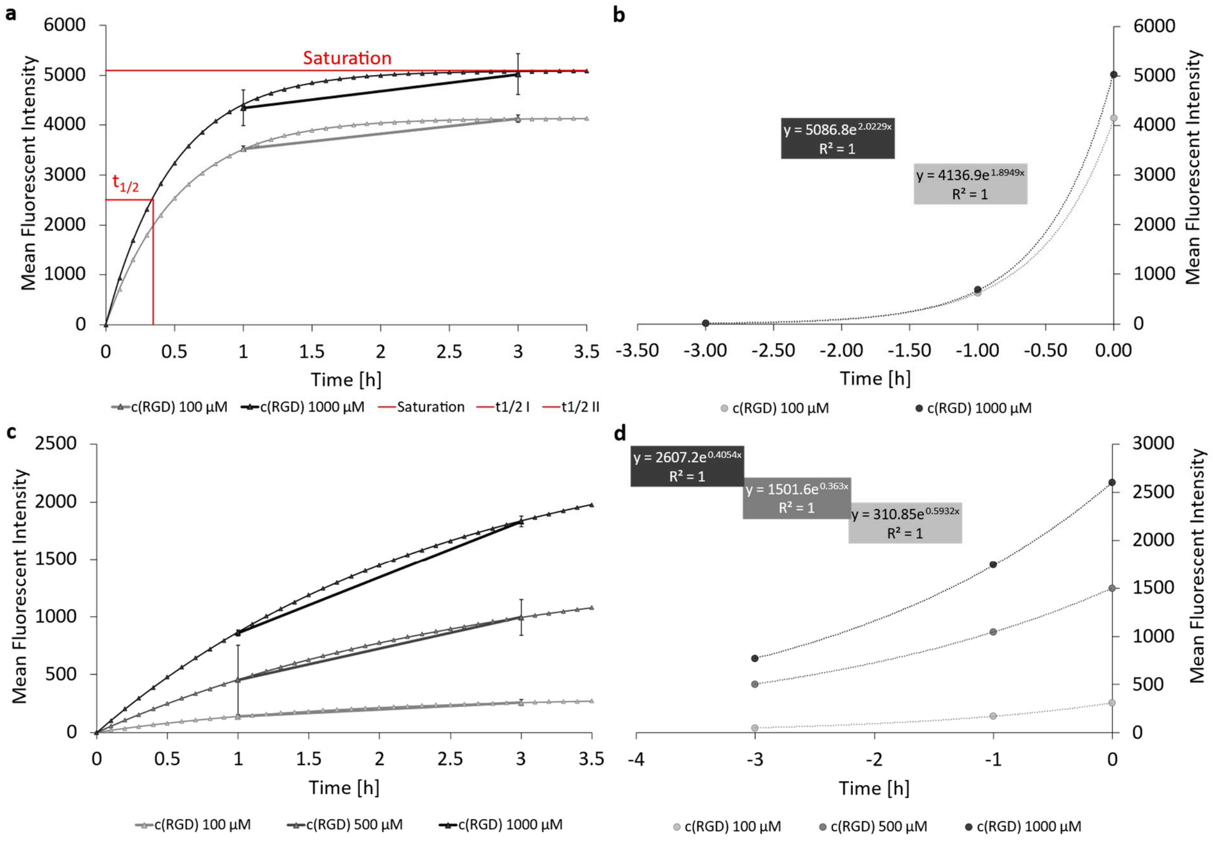

Figure S7: Plot of the mean fluorescence intensities over time (a and c) and the corresponding exponential fits (b and d) for the cellular uptake of c(RGD)-modified liposomes into the different glioblastoma cells, where. (a and b) represent the data for the U-87 MG cells and, (c and d) for the F98 cells.
